# Development of bone alkaline phosphatase-specific monoclonal antibodies and immunoassay exhibiting low cross-reactivity to liver isoform

**DOI:** 10.1093/jbmrpl/ziag080

**Published:** 2026-04-27

**Authors:** Tatsuji Kimura, Shiomi Ojima, Kosuke Doi

**Affiliations:** Diagnostic Division, Yamasa Corporation, Choshi, 288-0056, Japan; Diagnostic Division, Yamasa Corporation, Choshi, 288-0056, Japan; Diagnostic Division, Yamasa Corporation, Choshi, 288-0056, Japan

**Keywords:** alkaline phosphatase, isoform, glycosylation, bone turnover markers, bone formation, monoclonal antibody, immunoassay, epitope

## Abstract

Tissue-nonspecific alkaline phosphatase (TNSALP) is an enzyme that catalyzes the hydrolysis of phosphomonoesters and promotes mineralization of bone. Tissue-nonspecific alkaline phosphatase contains 5 N-glycosylation sites: N140, N230, N271, N303, and N430. Bone alkaline phosphatase (BALP) and liver alkaline phosphatase (LALP) are isoforms of TNSALP. Despite sharing identical amino acid sequences derived from the single gene *ALPL*, these isoforms exhibit different glycosylation patterns. As osteoblasts express BALP, serum BALP levels reflect osteoblastic activity and are used as markers of bone formation. However, distinguishing serum BALP from coexisting LALP is challenging, as the 2 isoforms differ only in carbohydrate composition. Moreover, while BALP-specific immunoassays using BALP-selective mAbs are currently used in clinical laboratories, they exhibit significant cross-reactivity with LALP, highlighting the need for more selective assays for BALP. In the present study, we developed mAbs against BALP with minimal cross-reactivity to LALP. Using these mAbs, we generated an in-house BALP-specific immunoassay that exhibited high correlation with a clinically approved BALP test kit. Using a series of single N-glycosylation site mutants of TNSALP, we found that all 6 clones of BALP-selective mAbs recognized an epitope relevant to N303-linked glycans. These mAbs could facilitate the development of improved BALP assays with reduced cross-reactivity to LALP, enabling a more accurate diagnostic evaluation, particularly in patients with liver disease.

## Graphical Abstract

**Graphical Abstract:**
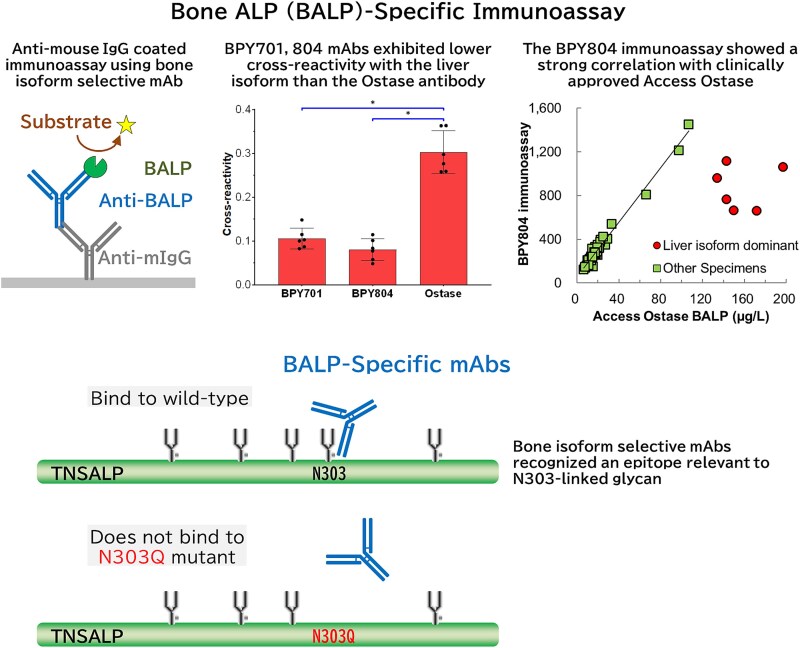

## Introduction

Alkaline phosphatase (ALP) (EC 3.1.3.1) is a dimeric enzyme that catalyzes the hydrolysis of phosphomonoesters. In humans, 4 ALP isozymes, namely intestinal ALP (IALP), placental ALP (PALP), germ cell ALP (GALP), and tissue-nonspecific ALP (TNSALP), are encoded by *ALPI*, *ALPP*, *ALPG*, and *ALPL*, respectively.^1^ Tissue-nonspecific ALP is a glycoprotein that plays key roles in bone formation and mineralization^1^^,^^2^ and is widely expressed across tissues and cells, with particular abundance in the bones, liver, and kidneys. Bone-ALP (BALP), liver-ALP (LALP), and kidney-ALP (KALP) are encoded by the same gene and are considered “isoforms” of the same “isozyme.” Although BALP and LALP share identical amino acid sequences, they differ in the post-translational modifications of their carbohydrate side chains. Each TNSALP monomer contains 5 potential N-glycosylation sites: N140, N230, N271, N303, and N430, all of which are fully occupied by N-glycans with varying degrees of sialylation.^3^ Notably, isoform differences are attributed to variations in glycosylation at these sites.^1^

Serum BALP, total or intact procollagen type I N-terminal propeptide (tP1NP or iP1NP, respectively), and osteocalcin (OC) are bone turnover markers (BTM) of bone formation. Among them, tP1NP can be measured using automated analyzers and is widely used in clinical settings. However, tP1NP levels are affected by renal dysfunction, whereas BALP levels remain independent of kidney function.^4–6^ Hence, BALP is particularly valuable for BTM, especially in patients with chronic kidney disease.^7^^,^^8^

Bone-ALP is produced by osteoblasts and anchored to the cell membrane via a glycosylphosphatidylinositol (GPI) anchor. It is released into the extracellular space via extracellular vesicles or as a soluble homodimer following the cleavage of the GPI anchor. Circulating ALP is predominantly composed of BALP and LALP in approximately equal proportions.^1^^,^^9^ However, selective quantification of BALP in serum is challenging, as it shares identical amino acid sequences to LALP, differing only in glycosylation patterns.^1^^,^^9^

Various techniques, such as heat inactivation, electrophoresis, and wheat germ lectin precipitation, have been developed to distinguish the glycosylation differences between BALP and LALP. However, their clinical utility has been limited by the complexity of the procedures.^10^ Moreover, while immunoassays using BALP-specific mAbs have enabled a broader range of clinical applications, they exhibit considerable cross-reactivity with LALP, potentially compromising BALP quantification in cases of elevated LALP.^11^^,^^12^ Thus, BALP assays with improved selectivity for the bone isoform (ie, reduced cross-reactivity with LALP) are urgently required.

In the present study, we developed mAbs against BALP with improved specificity to the bone isoform that could recognize an epitope associated with N303-linked glycans. Using these antibodies, we developed a BALP-selective immunoassay that exhibited a strong correlation with a clinically approved automated immunoassay.

## Materials and methods

### Animals

All animal experiments were approved by the Committee for Animal Experiments of the Yamasa Corporation (22-S-07, 23-S-06, 23-S-07, and 23-S-13). Female BALB/c mice (4 wk of age, 13-16 g) were obtained from Japan SLC (Hamamatsu, Japan) and allowed to acclimatize for 2 wk before immunization. Mice were group-housed under controlled conditions at a constant temperature (23 ± 3 °C) and humidity (50 ± 10%), a 12-h light/12-h dark cycle, and had ad libitum access to water and a standard rodent diet (CE-2; Clea Japan, Tokyo, Japan).

### mAb production

In the present study, Saos-2, a human osteosarcoma cell line that expresses high levels of BALP,^13^ was used as a source of the BALP immunogen, as previously described.^14^ BALB/c mice were intraperitoneally immunized with crude extract of Saos-2 cells (0.5-7.2 U of ALP activity, or equivalent to 5 × 10^6^ cells) emulsified in Freund’s complete adjuvant. Mice received booster immunization with the same antigen emulsified in Freund’s incomplete adjuvant 1-4 times at 2-10-wk intervals. Spleen cells were collected from mice 7 d after the last immunization and fused with SP2/O-Ag14 myeloma cells via the conventional polyethylene glycol method or electrofusion using a Super Electro Cell Fusion Generator (Nepa Gene, Ichikawa, Japan).^15^

Hybridomas in 96-well cell culture plates (Thermo Fisher Scientific, Waltham, MA, USA) were selected using hypoxanthine-aminopterin-thymidine, and an immunoassay was used to screen culture supernatants. Wells exhibiting reactivity to BALP derived from human serum were selected, and those demonstrating low cross-reactivity to LALP were prioritized. Hybridomas from selected wells were cloned by limiting dilution.

The subclass of mAbs was determined using sandwich ELISA (Jackson ImmunoResearch Laboratories, West Grove, PA, USA). Anti-mouse IgG (mIgG) was immobilized on 96-well microtiter plates (Iwaki, AGC Techno Glass, Shizuoka, Japan) to capture the mAbs. Horseradish peroxidase (HRP)-conjugated subclass-specific antibodies against anti-mIgG1, G2a, G2b, or G3 (Jackson ImmunoResearch Laboratories) were used for detection. Selected mAbs were purified from ascites fluid obtained from hybridoma-implanted mice using protein A affinity chromatography (Cytiva, Marlborough, MA, USA).

### Serum specimens and ALP isozymes

Serum specimens exhibiting high ALP levels were purchased from Precision for Medicine (Bethesda, MD, USA). Serum specimens with BALP or P1NP measurements were obtained from Central BioHub (Henningsdorf, Germany) and InVent Diagnostica (Berlin, Germany), respectively, via Central Link (Tokyo, Japan). All specimens were collected with informed consent.

Recombinant human IALP (rIALP) and human PALP were purchased from Cusabio (Houston, TX, USA) and Merck (Darmstadt, Germany), respectively.

### Clinically approved assays

ALP activity was measured using an L-Type Wako IFCC (Fujifilm Wako Pure Chemical, Osaka, Japan) on a TBA-120FR analyzer (Canon Medical Diagnostics Systems, Ohtawara, Tochigi, Japan). Bone alkaline phosphatase was measured using Access Ostase (Beckman Coulter, Brea, CA, USA).

The relative proportions (%) of each ALP isozyme were determined by electrophoretic analysis using Quick EP ALP-IF (Herena Laboratories, Saitama, Japan). In this system, ALP1 and ALP2 were classified as LALP, and ALP3 was considered BALP. The BALP/(BALP + LALP) ratio (B/BL ratio) was calculated using the following formula:


$$ \mathrm{B}/\mathrm{BL}\ \mathrm{ratio}=\mathrm{ALP}3\ (\%)/\left(\mathrm{ALP}1+\mathrm{ALP}2+\mathrm{ALP}3\right)\ (\%) $$

Specimens with a B/BL ratio ≥ 90% were categorized as BALP dominant, whereas those with a ratio < 10% were classified as LALP dominant.

### Evaluation of cross-reactivity to LALP

An anti-mIgG-coated immunoassay based on the enzymatic activity of BALP/LALP was used to evaluate the selectivity of mAbs. Briefly, 96-well white plates (MaxiSorp; Thermo Fisher Scientific) were coated with 100 μL/well of anti-mIgG (1 μg/mL) (Jackson ImmunoResearch Laboratories) diluted in Tris-buffered saline (TBS) containing 1 mM EDTA (EDTA-TBS). The wells were blocked with 1% bovine serum albumin (BSA)-EDTA-TBS for 1 h at 20-25 °C. After removal of the blocking solution, 50 μL of anti-BALP mAb (100 ng/mL) diluted in 1% BSA-EDTA-T-TBS was added to each well and incubated for 1 h at 20-25 °C. Subsequently, 50 μL of BALP- or LALP-dominant serum specimens diluted in 1% BSA-T-TBS was added to the wells and incubated for 1 h at 20-25 °C. After 3 washes with PBS containing 0.05% Tween 20 (T-PBS; standard wash buffer used in our laboratory), chemiluminescent substrate [disodium 2-chloro-5-(4-methoxyspiro {1,2-dioxetane-3,2′-(5′-chloro)tricyclodecan}-4-yl)-1-phenyl phosphate; CDP-Star with an Emerald II enhancer; Thermo Fisher Scientific] diluted in water (9:1:90 vol) was added and incubated for 20 min. The relative luminescence units (RLUs) were measured using a microplate reader (Tecan, Männedorf, Switzerland).

For comparison, the anti-BALP antibody employed in the Access Ostase (Beckman Coulter) kit (the Ostase antibody) was used without further purification. Our analysis indicated that the Ostase antibody belonged to the IgG2b subclass, with an IgG concentration of 6.8 μg/mL.

A panel of 6 BALP-dominant serum specimens with a B/BL ratio ≥ 90% and a panel of 6 LALP-dominant serum specimens with a B/BL ratio < 10% were used. All LALP-dominant serum specimens were obtained from specimens exhibiting high ALP activity. For the initial evaluation of 15 mAbs, designated BPY (from BALP Yamasa) series, serum specimens were diluted to an ALP activity of 10 U/L. Cross-reactivity to LALP was calculated as the signal obtained from LALP-dominant specimens divided by the mean signal from BALP-dominant specimens. The cross-reactivity values for each BPY mAb were compared with those of BPY508.

Antibody reactivity was assessed in serially diluted BALP-dominant or LALP-dominant serum specimens. Serum specimens were serially diluted to 8, 4, 2, and 1 U/L of ALP activity and measured using the anti-mIgG-coated ALP immunoassay with BPY508, BPY701, BPY804, or the Ostase antibody. To compare cross-reactivity with LALP, reaction levels (RLU) at an ALP concentration of 8 U/L were normalized to the mean reaction for the BALP-dominant serum specimens. Cross-reactivities to 6 LALP-dominant serum specimens were then compared among BPY mAs and the Ostase antibody.

### Correlation of BPY immunoassays with a clinically approved assay

A total of 64 commercially available serum specimens were used for this experiment. Among these, 8 were BALP-dominant, 6 were LALP-dominant, and the rest exhibited a B/BL ratio of 10%-90%. The assay method was identical to that used to evaluate cross-reactivity. Briefly, 96-well white plates were coated with anti-mIgG and blocked with BSA. After washing, 50 μL of BPY701 and BPY804 mAbs (100 ng/mL) in 1% BSA-EDTA-T-TBS was added to the wells and incubated for 1 h at 20-25 °C. Thereafter, 50 μL of serum specimens ×10 diluted in 1% BSA-T-TBS was added to the wells and incubated for an additional 1 h at 20-25 °C. After 3 washes, chemiluminescent substrate was added and incubated for 20 min, and the RLU values were measured.

### Preparation of N-glycosylation site mutants of TNSALP

N-glycosylation site mutants, as well as WT TNSALP and its recombinant expression, were designed as previously described.^3^^,^^16^ Briefly, plasmids encoding human TNSALP (UniProt: P05186, a.a, 1-489) and a series of single N-glycosylation site mutants were constructed. A FLAG (DYKDDDDK) epitope tag was introduced at the C-terminus of each TNSALP instead of the GPI-anchor region. The molecular weight of unmodified recombinant TNSALP (rTNSALP) was 54.7 kDa, as predicted by its amino acid sequence. The mammalian expression vector pYS-CMV-Puro (4427 bp), custom-synthesized by VectorBuilder (Yokohama, Japan), was used for expression. The coding sequences for TNSALP were cloned downstream of the cytomegalovirus (CMV) promoter, followed by an SV40 late polyadenylation signal. The vector also contains a puromycin resistance gene and an ampicillin resistance gene.

Saos-2 (1.3 × 10^5^ cells/well), human embryonic kidney cell line (HEK293; 2.5 × 10^5^ cells/well), human hepatoblastoma cell line (HepG2;2.5 × 10^5^ cells/well), and African green monkey kidney fibroblast-like cell line (COS-7; 2.5 × 10^5^ cells/well) cells were cultured in 6-well plates and transfected with 2.5 μg of each plasmid vector using Lipofectamine 3000 Reagent (Invitrogen, Carlsbad, CA, USA) following the manufacturer’s protocol. Transfected cells were incubated for 4, 2, 4, or 2 d, respectively, in a 5% CO_2_ incubator. Culture supernatants were then collected for subsequent analysis.

### Western blot

Culture supernatants containing rTNSALP-FLAG (WT, Mutants) were mixed with 2-mercaptoethanol-containing SDS-PAGE sample buffer and treated at 99 °C for 5 min. Samples (10 or 5 μL of culture supernatant from Saos-2 or HEK293 cells, respectively) were separated by electrophoresis on a 4%-20% polyacrylamide gel (Mini-PROTEAN TGX; BioRad Laboratories, Hercules, CA, USA) at 150 V for 35 min and transferred to a polyvinylidene difluoride membrane (Immobilon-E, 0.45 μm; Millipore, Billerica, MA, USA) using a semi-dry blotter (Trans-Blot Turbo Transfer System; BioRad Laboratories). Membranes were blocked with EDTA-TBS containing 0.5% casein (Sigma-Aldrich, St. Louis, MO, USA) and then incubated at 20-25 °C for 1 h with HRP-conjugated anti-FLAG rabbit mAb (D6W5B; Cell Signaling Technology, Danvers, MA, USA) or anti-TNSALP rabbit polyclonal antibody (Proteintech, Rosemont, IL, USA). For detection with the anti-TNSALP antibody, an additional incubation with HRP-conjugated anti-rabbit IgG (Jackson ImmunoResearch Laboratories) was performed. Membranes were washed thoroughly with TBS containing 0.5% Tween 20, and HRP activity was detected using a chemiluminescent substrate (ImmunoStar Zeta; Fujifilm Wako Pure Chemical) and visualized using an Amersham Imager 680 (GE Healthcare, Chicago, IL, USA).

### Comparison of reactivity to the N-glycosylation mutants using sandwich ELISA

Three BALP-selective mAbs (BPY701, BPY703, and BPY804) and 3 nonselective mAbs (BPY402, BPY504, and BPY805) were conjugated with HRP using a Peroxidase Labeling Kit-NH_2_ (Dojindo, Kumamoto, Japan). Subsequently, 96-well ELISA plates (Thermo Fisher Scientific) were treated with 50 μL/well of anti-FLAG mAb (anti-DYKDDDDK tag, 5 μg/mL; Fujifilm Wako Pure Chemical) in EDTA-TBS. The wells were then blocked with EDTA-TBS containing 1% BSA (SeraCare, Milford, MA, USA) and subsequently washed. Culture supernatant containing rTNSALP-FLAG (WT and mutants) from Saos-2, HEK293, HepG2, or COS-7 cells was diluted to ×2, ×10, ×5, or ×5, respectively, in 1% BSA-T-TBS containing 1 mM MgCl_2_ and 0.1 mM ZnCl_2_. A 50-μL aliquot of each diluted sample was added to the wells and incubated for 1 h at 37 °C. Subsequently, 50 μL of HRP-conjugated BPY mAb (0.005-1 μg/mL) in the same diluent was added to the wells and incubated for 1 h at 37 °C. The concentrations of the HRP-BPY mAbs were 0.005, 1, 0.2, 0.5, 0.2, and 0.3 μg/mL for HRP-BPY805, HRP-BPY402, HRP-BPY504, HRP-BPY701, HRP-BPY703, and HRP-BPY804, respectively, to allow comparison within a similar range of reaction intensity. After 3 washes with T-PBS, 100 μL/well of the colorimetric substrate (3,3′,5,5′-tetramethylbenzidine/H_2_O_2_) was added and incubated for 15 min, and the reaction was stopped by adding 50 μL/well of H_2_SO_4_ (0.5 M). Absorbance was measured at 450 nm using a microplate reader (Tecan).

### Comparison of reactivity to the N-glycosylation mutants using anti-mIgG-coated ALP immunoassay

This assay was conducted following a protocol similar to that used for evaluating cross-reactivity. Briefly, 96-well white plates were coated with anti-mIgG and blocked with BSA. After washing, 50 μL of BPY series mAb (1 μg/mL) was added to the wells and incubated for 1 h at 20-25 °C. Subsequently, 50 μL of rTNSALPs (WT and mutants) diluted to an ALP activity of 2 U/L was added to the wells and incubated for 1 h at 20-25 °C. Following 3 washes, chemiluminescent substrate was added and incubated for 20 min, and the RLU values were measured.

### Statistical analysis

To compare cross-reactivity with LALP among the antibodies, a repeated-measures ANOVA (RM-ANOVA) was first performed to evaluate the overall effect of antibody type. Cross-reactivity values for each antibody were further compared with a pre-specified baseline (BPY508 or the Ostase antibody) using paired *t*-tests with Holm adjustment for multiple comparisons. A *p*-value of <.05 was considered statistically significant.

Regression lines were calculated using the least-squares method. The correlation between the BPY immunoassay and the Access Ostase antibody was assessed using the Pearson correlation coefficient. All statistical analyses were performed using JMP Pro software version 18.2.2 (SAS Inc., Cary, NC, USA).

## Results

### mAbs against BALP

We performed cell fusion experiments 8 times. In the first screening, 1%-41% of seeded wells (46/940, 10/994, 78/188, 14/1034, 42/1504, 40/1410, 51/1380, and 23/1380 from fusion 1-8, respectively) were positive for BALP reactivity. Then, we prioritized wells with low cross-reactivity to LALP for subsequent cloning. Finally, we established 28 hybridoma clones secreting BALP-reactive mAbs, designated the BPY (from *B*AL*P Y*amasa) series. From these clones, we selected 14 mAbs for further analysis; 8 were expected to be BALP-selective, and the remaining 6 were designated as nonselective controls. We subsequently purified these mAbs.

### Cross-reactivity to LALP

**Figure 1:**
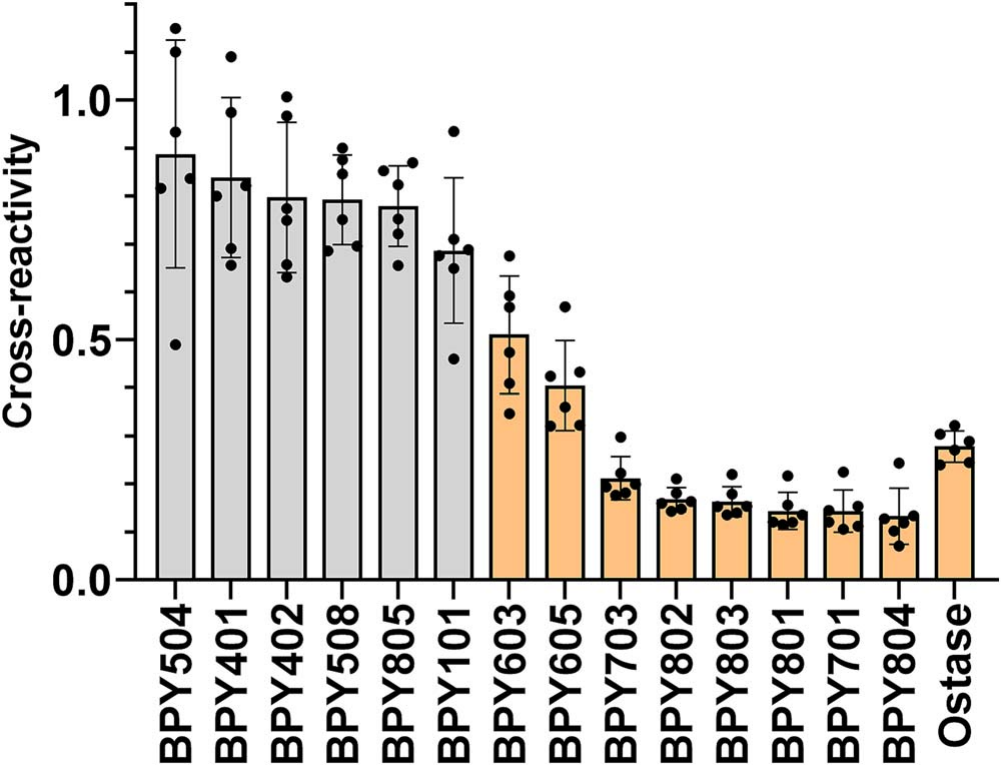
Cross-reactivity to liver alkaline phosphatase (LALP). Cross-reactivity to LALP was individually calculated for each of the 14 BPY antibodies and the Ostase antibody, based on the results presented in Table 1, as the reaction to each LALP-dominant specimen (10 U/L) divided by the mean reaction of the BALP-dominant specimens (10 U/L). Cross-reactivities obtained using anti-mouse IgG-coated immunoassays are presented as bars with overlaid individual data points. Error bars represent the mean ± SD. Differences among antibodies were evaluated by comparing the cross-reactivity values for each BPY antibodies with those of BPY508 using paired *t*-tests as planned contrasts with Holm adjustment (Table S1). Antibodies exhibiting significant differences in cross-reactivity relative to BPY508 (*p* < .05) are highlighted in pale orange.

We evaluated the reactivities of the 14 mAbs to BALP-dominant or LALP-dominant serum specimens using an immunoassay based on the activity of BALP/LALP (Table 1). We calculated cross-reactivity with LALP as the relative reaction level normalized to the mean reaction level of BALP-dominant specimens (Table 1, Figure 1). Six mAbs (BPY504, BPY401, BPY402, BPY508, BPY805, and BPY101), which exhibited cross-reactivity ranging from 88.8% to 68.6%, were considered lacking significant selectivity between BALP and LALP. We compared cross-reactivity with 6 LALP-dominant specimens (10 U/L) among the 14 BPY mAbs and the Ostase antibody. RM-ANOVA revealed a significant main effect of antibody difference (F(14, 70) = 55.5, *p* < .001). Planned contrasts using BPY508 as the typical nonselective antibody baseline demonstrated that the BPY603, BPY605, BPY703, BPY802, BPY803, BPY801, BPY701, BPY804, and the Ostase antibody differed significantly from BPY508 after Holm correction (*p* < .001). Complete results for all 14 planned contrasts versus BPY508 are provided in Table S1. The RLU values varied depending on the mAb. BALP-selective mAbs generally exhibited lower overall activity than other mAbs (Table 1).

**Table 1:** Anti-bone alkaline phosphatase (BALP) mAbs and their reactivity to BALP and liver alkaline phosphatase (LALP).

**Antibody**	**Subclass**	**BALP dominant (RLU)**	**LALP dominant (RLU)**	**Cross-reactivity to LALP**
**BPY504**	IgG1	579 299 ± 247 236	514 307 ± 137 641	0.888 ± 0.238
**BPY401**	IgG1	841 270 ± 203 277	705 924 ± 140 304	0.839 ± 0.167
**BPY402**	IgG1	739 930 ± 95 828	590 068 ± 116 068	0.797 ± 0.157
**BPY508**	IgG1	967 919 ± 39 264	767 035 ± 90 254	0.792 ± 0.093
**BPY805**	IgG2a	1 146 285 ± 60 202	893 003 ± 96 101	0.779 ± 0.084
**BPY101**	IgG1	262 176 ± 31 836	179 877 ± 39 757	0.686 ± 0.152
**BPY603**	IgG1	600 267 ± 113 415	306 580 ± 73 684	0.511 ± 0.123
**BPY605**	IgG1	507 499 ± 69 811	205 609 ± 47 403	0.405 ± 0.093
**BPY703**	IgG1	96 693 ± 15 211	20 527 ± 4324	0.212 ± 0.045
**BPY802**	IgG1	85 560 ± 10 246	14 355 ± 2143	0.168 ± 0.025
**BPY803**	IgG1	115 663 ± 13 907	18 918 ± 3618	0.164 ± 0.031
**BPY801**	IgG1	73 330 ± 12 979	10 571 ± 2857	0.144 ± 0.039
**BPY701**	IgG1	71 549 ± 11 639	10 290 ± 3140	0.144 ± 0.044
**BPY804**	IgG2b	203 761 ± 61 220	27 121 ± 12 035	0.133 ± 0.059
**Ostase**	IgG2a	78 893 ± 11 372	21 974 ± 2576	0.279 ± 0.033

Abbreviations: BALP, bone alkaline phosphatase; LALP, liver alkaline phosphatase; RLU, relative luminescence units.

Reactivity of anti-BALP mAbs (100 ng/mL) to each of the 6 specimens of BALP- and LALP-dominant sera (10 U/L of alkaline phosphatase activity) was measured using an anti-mouse IgG-coated immunoassay. Results are presented as the mean ± SD of 6 BALP-dominant serum specimens (B/BL ratio ≥ 90%) and 6 LALP-dominant serum specimens (B/BL ratio < 10%). Cross-reactivity to LALP was individually calculated for each of the 14 BPY mAbs and the Ostase antibody as the reaction to each LALP-dominant specimen divided by the mean reaction of the BALP-dominant specimens.

Moreover, we analyzed isozyme specificity using a similar approach. All 14 mAbs demonstrated cross-reactivity of <0.04% and < 0.4% with rIALP and PALP, respectively (Table S2). Thus, we considered these 14 mAbs to be “TNSALP isozyme-specific,” including 8 “bone isoform-selective” mAbs.

We also compared the cross-reactivities of BPY mAbs with that of the Ostase antibody using serially diluted BALP-dominant and LALP-dominant serum specimens (*n* = 6 each) (Figure S1). In an anti-mIgG-coated ALP immunoassay, BPY508, BPY701, BPY804, and the Ostase antibody all exhibited linear, dilution-dependent decreases in reaction levels. Notably, BPY701, BPY804, and the Ostase antibody demonstrated lower reactivity toward LALP-dominant serum specimens than toward BALP-dominant serum specimens, whereas BPY508 exhibited similar reactivity toward both specimen types (Figure S1).

**Figure 2:**
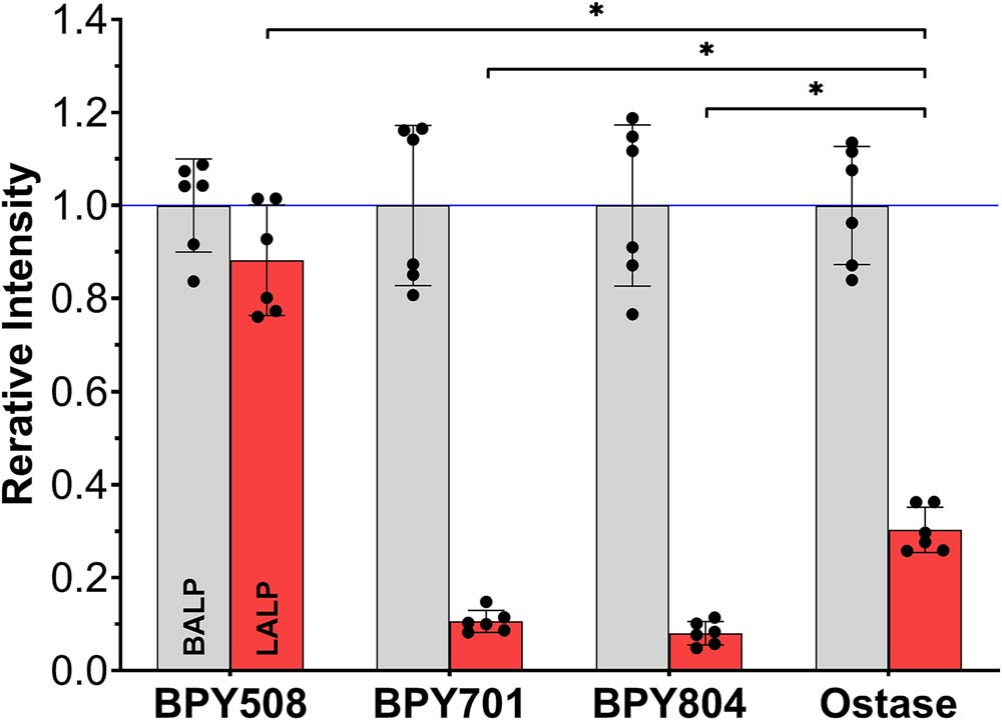
Bone alkaline phosphatase (BALP) and liver alkaline phosphatase (LALP) reactivity. Six BALP-dominant (B/BL ≥ 90%) and 6 LALP-dominant (B/BL < 10%) serum specimens were diluted to 8 U/L of ALP activity, and their reactivity was evaluated using anti-mouse IgG-coated immunoassays with BPY508, BPY701, BPY804, or the Ostase antibody. Each immunoassay was performed in duplicate, and the mean value was used for analysis. To compare the relative reactivity across antibodies, the RLU values for each of the 4 antibodies were normalized independently by setting the mean value of the 6 BALP-dominant serum specimens to 1 in each assay. The relative intensities are presented as bars with overlaid individual data points, and the error bars represent the mean ± SD. The cross-reactivities (relative intensities for LALP) of the BPY antibodies were compared with the Ostase antibody using paired *t*-tests as planned contrasts with holm adjustment. Significant differences (*p* < .05) are indicated by brackets with asterisks. RLU, relative luminescence units.

To characterize these differences statistically, we normalized the reaction levels (RLU) at an ALP concentration of 8 U/L to the mean value obtained from the BALP-dominant serum specimens for each antibody assay and assessed cross-reactivity among the antibodies (Figure 2). The RM-ANOVA revealed a significant main effect of antibody difference (F(3, 15) = 276, *p* < .001). The calculated LALP cross-reactivities (mean ± SD) were as follows: BPY508 (0.882 ± 0.119), BPY701 (0.106 ± 0.024), BPY804 (0.081 ± 0.025), and the Ostase antibody (0.303 ± 0.049). Planned contrasts using the Ostase antibody as baseline, BPY701 (mean difference = −0.197, 95% CI: −0.232, −0.161, Holm-adjusted *p* < .001) and BPY804 (mean difference = −0.222, 95% CI [−0.268, −0.176], Holm-adjusted *p* < .001) exhibited significantly lower cross-reactivity than the Ostase antibody.

### BPY immunoassay for serum specimens and correlations to access Ostase

**Figure 3:**
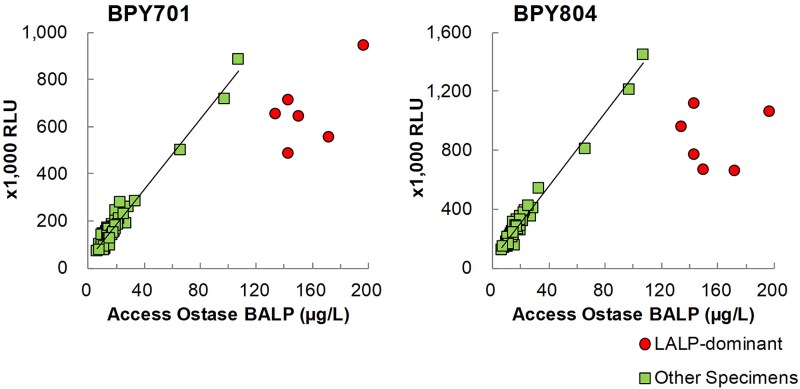
Correlation with the access Ostase antibody. Bone alkaline phosphatase (BALP) was measured using anti-mouse IgG-coated immunoassays with BPY701 and BPY804 (RLU) and the access Ostase clinical assay (μg/L) in serum specimens (*n* = 64), comprising 6 liver ALP (LALP)-dominant serum specimens (B/BL ratio < 10%) and 58 other serum specimens (B/BL ratio ≥ 10%). Each immunoassay was performed in duplicate, and the mean value was used for analysis. Regression analyses were performed using the 58 serum specimens excluding the LALP-dominant specimens. The regression equations were as follows: BPY701 vs access Ostase: *y* = 7527*x* + 30 342, *r* = 0.983; BPY804 vs access Ostase: *y* = 12 483*x* + 54 760, *r* = 0.989. RLU, relative luminescence units.

We developed a microplate-based in-house immunoassay (BPY immunoassay) to measure BALP levels in serum specimens. We compared the results of the BPY immunoassays using BPY701 or BPY804 with those of a clinically approved Access Ostase assay using 64 commercial serum specimens, comprising 8 BALP-dominant, 6 LALP-dominant, and the other 50 serum specimens (Figure 3). Our results indicated that BPY701 and BPY804 immunoassays exhibited a strong correlation with Access Ostase (*r* = 0.983 and 0.989, respectively) in most specimens, except for LALP-dominant specimens, wherein the reactive intensities of the immunoassays were plotted below the regression line.

### Epitope analysis of mAbs

We investigated the epitopes recognized by the BPY mAbs. Epitope mapping using synthesized short peptides is a standard approach for identifying linear epitopes of mAbs. As a prerequisite for peptide-based mapping, we first examined whether the BPY mAbs could recognize denatured TNSALP. Western blot analysis of Saos-2 cell lysates revealed that BPY402 detected a clear band corresponding to TNSALP, whereas the BALP-selective mAbs (BPY701 and BPY703) demonstrated no detectable reactivity (data not shown). These findings indicate that the BALP-selective antibodies do not bind denatured TNSALP and are thus unlikely to recognize linear peptide epitopes. Consequently, we did not pursue peptide-based epitope mapping for these antibodies.

Based on our findings, we hypothesized that BALP-selective mAbs recognize glycans that differ between BALP and LALP. To test this hypothesis, we generated WT rTNSALP and a series of single N-glycosylation site mutants, each tagged with a FLAG epitope at the C-terminus. The rTNSALP-FLAG constructs were expressed in Saos-2, HEK293, HepG2, and COS-7 cells. The types of mutations and corresponding ALP activities in the culture supernatant are listed in Table 2. We observed high activity derived from overexpressed TNSALP proteins in WT and 5 mutants, except for N430Q, which lacked ALP activity. This result is consistent with a previous report demonstrating that the N430Q mutant prevents formation of the dimeric structure required for ALP activity.^16^ ALP activity levels in the culture supernatant of transfected HEK293 cells were 70-138-fold higher than those in the supernatant of transfected Saos-2 cells.

**Table 2:** N-glycosylation-site mutants of tissue-nonspecific alkaline phosphatase (TNSALP) and their alkaline phosphatase (ALP) activity.

**#**	**Type**	**Predicted glycosylation**	**ALP (U/L)**			
			**Saos-2**	**HEK293**	**HepG2**	**COS-7**
**1**	Mock		62 ± 5	14 ± 8	18	7
**2**	WT	5 sites	323 ± 81	29 250 ± 7071	2570	2465
**3**	N140Q	ΔN140	370 ± 42	25 900 ± 4313	560	1145
**4**	N230Q	ΔN230	318 ± 95	28 025 ± 11 773	1710	1670
**5**	N271Q	ΔN271	145 ± 35	19 950 ± 8839	745	1385
**6**	N303Q	ΔN303	365 ± 92	27 075 ± 14 602	1605	1270
**7**	N430Q	ΔN430	54 ± 13	14 ± 7	16	8
**8**	N430D	ΔN430	325 ± 57	29 775 ± 7814	2710	2465

Abbreviations: ALP, alkaline phosphatase; TNSALP, tissue-nonspecific alkaline phosphatase.

Saos-2, HEK293, HepG2, or COS-7 cells were transfected with TNSALP (WT or mutants). ALP activity in the culture supernatant was measured in duplicate, and the mean value was used for analysis. The results for Saos-2 and HEK293 cells are presented as the mean ± SD from 2 independent expression experiments. ALP activities obtained from single expression experiments in HepG2 and COS-7 cells are also presented.

**Figure 4:**
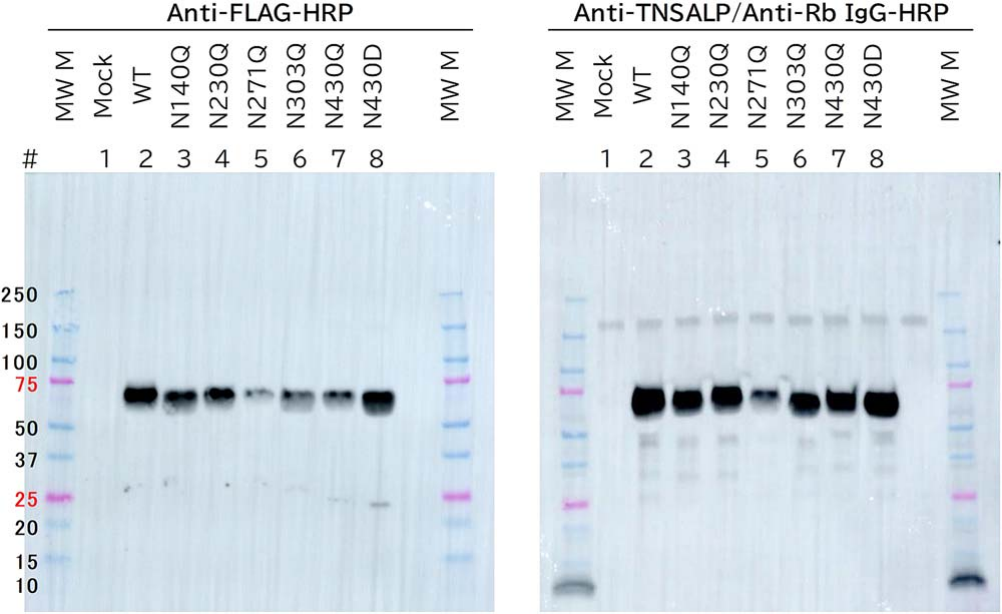
Western blot analysis of N-glycosylation-site mutants of tissue-nonspecific alkaline phosphatase (TNSALP). WT and N-glycosylation-site mutant TNSALP proteins expressed in HEK293 cells were analyzed by western blotting. Each 5 μL of the recombinant TNSALP-expressing culture supernatants was subjected to electrophoresis. The left panel presents results obtained using the anti-***FLAG antibody, while the right panel presents results obtained with the anti-TNSALP antibody.

We confirmed protein expression using western blot analysis (Figure 4). In HEK293 cells, both anti-FLAG and anti-TNSALP antibodies detected 70 kDa bands corresponding to TNSALP-FLAG in the WT and all 6 mutants. The band intensity for the N271Q was weaker than that of the other constructs, consistent with its decreased ALP activity (Table 2). Although the N430Q protein was expressed, no ALP activity was observed, consistent with a previous report.^16^ In Saos-2 cells, the 70 kDa bands corresponding to TNSALP-FLAG were faint or undetectable (data not shown), likely owing to decreased expression levels (Table 2). The N271Q expression level in Saos-2 cells was assumed to be lower than that of the other mutants, consistent with ALP activity (Table 2).

**Figure 5:**
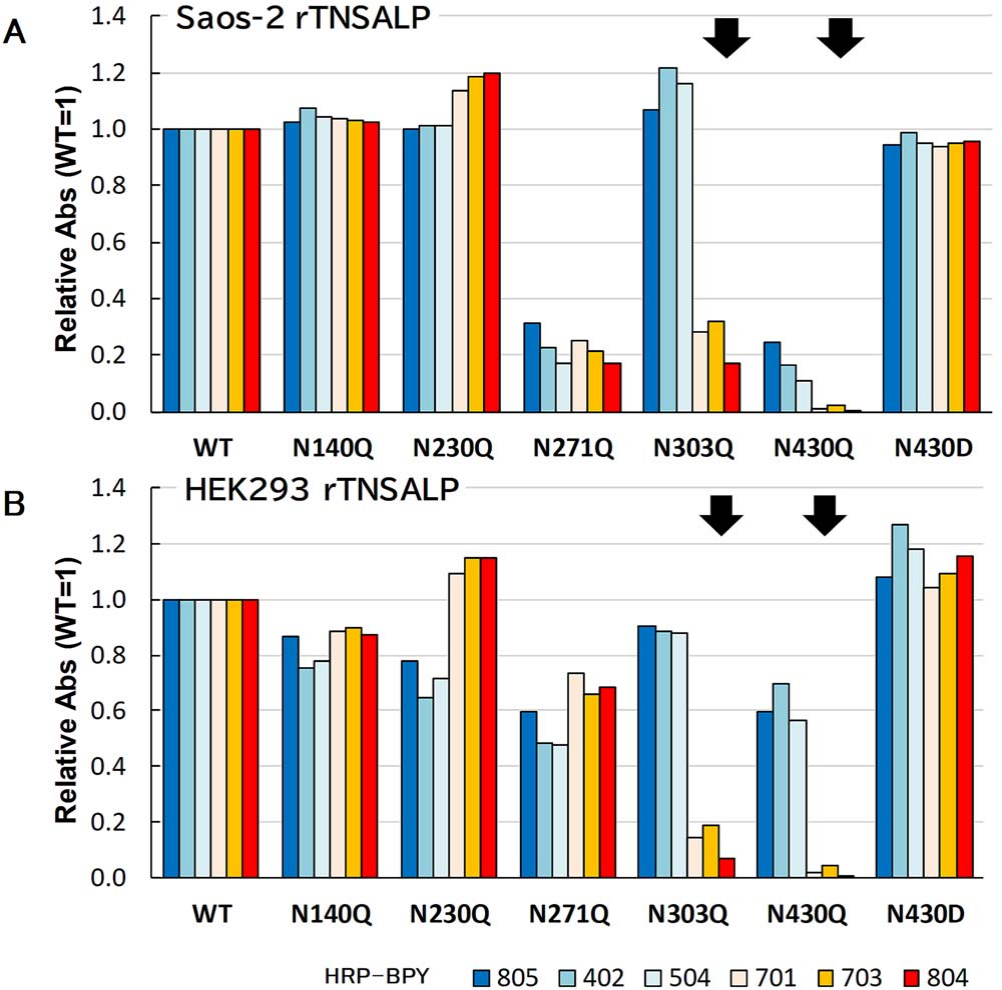
Reactivity to N-glycosylation site mutants assessed by sandwich ELISA. Recombinant tissue-nonspecific alkaline phosphatase (rTNSALP) in culture supernatants was measured by sandwich ELISA with anti-FLAG capture antibody and horseradish peroxidase-conjugated BPY detection antibodies. Reaction intensities are expressed as relative absorbance values normalized to WT (WT = 1), as indicated on the *y*-axis [“Relative Abs (WT = 1)”]. (A) Culture supernatant from Saos-2 cells (2× dilution). (B) Culture supernatant from HEK293 cells (10× dilution). Arrows indicate reduced reactivity attributable to the mutations.

We also assessed the reactivity of the mAbs to rTNSALP-FLAG (WT and mutants) using sandwich ELISA (Figure 5). The nonselective mAbs (BPY402, BPY504, and BPY805) exhibited comparable reactivity to WT and mutants N140Q, N230Q, N303Q, and N430D, but reduced reactivity to N271Q and N430Q, particularly in Saos-2 cells, likely reflecting decreased protein expression levels. Reactivity to N140Q, N230Q, N271Q, and N430D was similar across all 6 mAbs. In contrast, BALP-selective mAbs (BPY701, BPY703, and BPY804) demonstrated lower reactivity to N303Q and N430Q than the nonselective mAbs (BPY402, BPY504, and BPY805) in both Saos-2 and HEK293 cells. We observed similar results in HepG2 and COS-7 cells (Figure S2). Collectively, these results suggest that BALP-selective mAbs (BPY701, BPY703, and BPY804) recognize epitopes associated with N303-linked glycans on the dimeric form of TNSALP.

**Figure 6:**
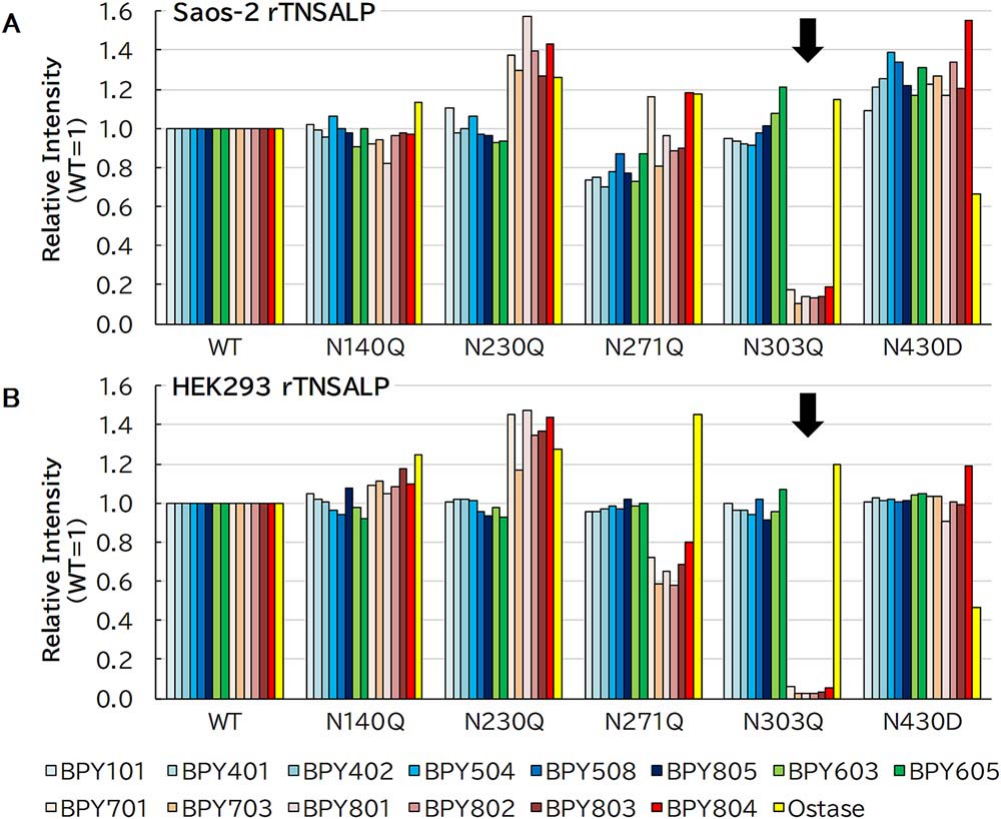
Reactivity to N-glycosylation site mutants assessed by anti-mouse IgG-coated immunoassay recombinant tissue-nonspecific alkaline phosphatase (rTNSALP)-expressing culture supernatants (2 U/L of ALP activity) were analyzed using anti-mouse IgG-coated immunoassay. The mean reaction intensities (RLUs) of duplicate assays are expressed as relative values normalized to the WT (WT = 1), as indicated on the *y*-axis [“Relative Intensity (WT = 1)”]. (A) Expression in Saos-2 cells. (B) Expression in HEK293 cells. Arrows indicate reduced reactivity attributable to the mutation.

We further examined the reactivity of the mAbs using an anti-mIgG-coated ALP immunoassay (Figure 6), which does not require purification, labeling, or a large quantity of antibodies. This allowed assessment of the reactivity of the 14 BPY mAbs and the Ostase antibody. However, the immunoassay failed to distinguish between the recombinant and endogenous TNSALP. To reduce background associated with the high endogenous TNSALP in Saos-2 cells, we also expressed TNSALP in HEK293 cells, which exhibits very low endogenous TNSALP expression. In this assay, we diluted recombinant TNSALPs (WT and mutant) to an identical ALP activity of 2 U/L. However, we did not assess the inactive N430Q mutant, as it could not be evaluated using this ALP immunoassay. The reactivity of BPY mAbs to the 4 N-glycosylation site mutants (N140Q, N230Q, N271Q, and N430D), except N303Q, was comparable to that observed for the WT for all BPY mAbs (Figure 6). The nonselective (BPY101, BPY401, BPY402, BPY504, BPY508, and BPY805) and low-selective (BPY603 and BPY605) mAbs also reacted with the N303Q mutant. BALP-selective mAbs (BPY701, BPY703, BPY801, BPY802, BPY803, and BPY804) exhibited strikingly lower reactivity to the N303Q mutant than the WT in Saos-2 cells, and almost no reactivity in HEK293 cells. In contrast, the antibody from the Access Ostase kit demonstrated reduced reactivity to N430D but did not exhibit a complete loss of signal, even in HEK293 cells (Figure 6). We observed a similar result in HepG2 cells (Figure S3).

Collectively, these results suggest that our BALP-selective BPY mAbs recognize an epitope containing an N-glycan modification at N303, whereas the Ostase antibody likely targets an epitope associated with the N430-linked glycan.

## Discussion

The increasing availability of commercial immunoassays for BALP has substantially expanded its use as a BTM. In 1989, Hill and Wolfert reported 5 mAbs with high specificity for BALP,^14^ prompting the development of the first commercially available immunoradiometric assay, the Tandem-R Ostase (Hybritech; now Beckman Coulter). This initial platform was subsequently complemented by microplate-based alternatives, including Tandem-MP Ostase (Beckman Coulter), in which a single mAb (BA1G017, IgG2a) was used,^10^^,^^17^ and Alkphase-B (Metra Biosystems; now Quidel, San Diego, CA, USA).^18^^,^^19^ Since then, different automated immunoassays for BALP measurement have been proposed, including Access Ostase (Beckman Coulter), iSYS Ostase (Immunodiagnostic Systems, Boldon, UK), and LIAISON BAP Ostase (DiaSorin, Saluggia, Italy).^20^ However, the clinical reliability of these assays is limited by cross-reactivity with LALP, which is reportedly 7%-18% for Tandem-R and Tandem-MP Ostase, and 3%-15% for Alkphase-B,^10^ potentially leading to falsely elevated BALP levels in patients with liver disease. Therefore, a BALP assay with decreased cross-reactivity to LALP is required.

In the present study, we established bone isoform-selective mAbs with low cross-reactivity to the liver isoform. Our mAbs exhibited lower cross-reactivity with LALP than the antibody of the clinically approved Access Ostase kit (Figure 2). Notably, our BALP assay using BPY804 or BPY701 mAbs demonstrated high correlation with Access Ostase (Figure 3). These mAbs hold considerable promise for developing an improved BALP measurement with reduced cross-reactivity to LALP, which would enable more accurate diagnostic evaluation, particularly in patients with liver failure.

Moreover, a mutation at the N303 glycosylation site results in loss of reactivity of BALP-selective mAbs with LALP, as demonstrated by sandwich ELISA (Figures 5 and S2) and anti-mIgG-coated ALP immunoassay (Figures 6 and S3). TNSALP undergoes modification at 5 N-glycosylation sites with different sugar chain compositions. As Saos-2 is a human osteosarcoma cell line, rTNSALP exogenously expressed in Saos-2 cells was regarded equivalent to BALP, as was endogenous TNSALP. However, our anti-mIgG-coated ALP immunoassay could not distinguish between endogenous and recombinant BALP proteins, whether WT or mutant variants. We considered the residual reactivity of BALP-selective BPY mAbs with the N303Q mutant expressed in Saos-2 cells (Figure 6A) to be attributed to cross-reactivity with endogenous (WT) BALP. This interpretation was supported by parallel expression experiments in HEK293 and HepG2 cells (Figures 6B and S3), which exhibited decreased endogenous TNSALP expression compared to Saos-2 cells (Table 2, Mock). This reduced background signal rendered negligible. Notably, the rTNSALP expressed in HEK293 cells cannot be considered the bone isoform (BALP), as HEK293 is a human embryonic kidney cell line.

We observed similar results in HepG2 (human hepatoblastoma) and COS-7 cells (African green monkey kidney fibroblast-like cells) (Figures S2 and S3). While we initially expected that rTNSALP expressed in HepG2 cells would exhibit liver-type characteristics, the reactivity profiles of the BALP-selective antibodies (Figures S2A and S3) were comparable to those observed for rTNSALP expressed in Saos-2 cells or HEK293 cells (Figures 5 and 6). A previous study indicated that HepG2 cells aberrantly glycosylated TNSALP.^21^ Consequently, rTNSALP overexpressed in HepG2 cells may not recapitulate liver-type glycosylation patterns. Although our overexpression studies clearly indicate that N-glycosylation at position 303 affects antibody reactivity, they do not allow us to distinguish differences in glycosylation patterns between bone-type and liver-type TNSALP.

Collectively, these results indicate that the mAbs BPY701, BPY703, BPY801, BPY802, BPY803, and BPY804 fail to recognize the N303Q glycan-deletion mutant. Therefore, these mAbs likely recognize N303-linked glycans, either the glycan epitopes or the glycans and their surrounding regions of the protein. However, we cannot definitively exclude the possibility that these mAbs recognize a bone isoform-specific conformational epitope affected by N303-linked glycans. While we attempted to explore mAb reactivity to N-glycosidase-treated TNSALP, we obtained a substantially diminished signal in our ALP immunoassay. This finding is consistent with previous reports indicating that cleavage of N-glycosylation leads to complete loss of activity.^16^^,^^22^ Therefore, the glycan structure recognized by these antibodies remains to be elucidated.

BALP-selective mAbs have been thought to recognize epitopes dependent on glycan structures distinct from those present on LALP. However, in the International Society of Oncodevelopmental Biology and Medicine Tissue Differentiation Workshop 9 (ISOBM TD9),^10^ a specific recognition site for BALP-selective mAbs could not be localized using a combination of cross-inhibition and mutation experiments. In this context, we investigated epitopes associated with N303-linked glycans using glycosylation site mutants, thereby providing a complementary mechanistic perspective. While the specific differences in oligosaccharide structures between BALP and LALP remain unknown, mAbs specific to a particular glycan structure, including our BALP-selective mAbs, may serve as valuable tools for elucidating the significance of glycosylation. Furthermore, the finding that the Ostase antibody, which targets N430-linked glycans, can distinguish BALP from LALP suggests that glycosylation at N430 differs between the 2 isoforms. In addition, differences in O-linked glycosylation have been reported,^22^ and the physiological significance of the heterogeneous modifications of TNSALP remains uncertain and warrants further investigation. Overall, the BPY mAbs (recognizing N303) and the antibody used in the Access Ostase kit (targeting N430) may serve as powerful tools for future studies on TNSALP.

BALP comprises 4 subpopulations (BALP isoforms: B/I, B1x, B1, and B2) that can be separated using high-performance liquid chromatography.^23^^,^^24^ Among them, serum B1x is exclusively present in patients with chronic kidney disease and is associated with prolonged survival, highlighting its potential as a biomarker for clinical use.^24^^,^^25^ However, current immunoassays for BALP cannot differentiate between BALP isoforms.^3^^,^^24^^,^^25^ Therefore, determining whether our BPY mAbs can distinguish among these BALP isoforms is of considerable clinical interest and will be the subject of future studies.

However, this study is limited by the small number of commercial serum specimens used, warranting further validation using clinical serum specimens. In addition, the BALP assays used in this study were preliminary, manual, in-house assays intended for laboratory research purposes. Therefore, the development of an automated BALP assay using these mAbs and its clinical applications are greatly anticipated.

Our bone isoform-selective mAbs pave the way for the development of improved BALP assays by effectively circumventing the long-standing challenge of LALP cross-reactivity. This advancement will refine diagnostic accuracy, particularly in patients with liver disease.

## Supplementary Material

Fig_S1_ziag080

Fig_S2_ziag080

Fig_S3_ziag080

Table_S1_ziag080

Table_S2_ziag080

## Data Availability

Data supporting the findings of this study are available from the corresponding author upon request.
