# Supplementary material for: Development of bone alkaline phosphatase-specific monoclonal antibodies and immunoassay exhibiting low cross-reactivity to liver isoform
Source: JBMR Plus. 2026 Apr 27;10(6):ziag080. doi: 10.1093/jbmrpl/ziag080 (PMC13184525; doi:10.1093/jbmrpl/ziag080)
Supplement: Fig_S1_ziag080 [file fig_s1_ziag080.pdf]

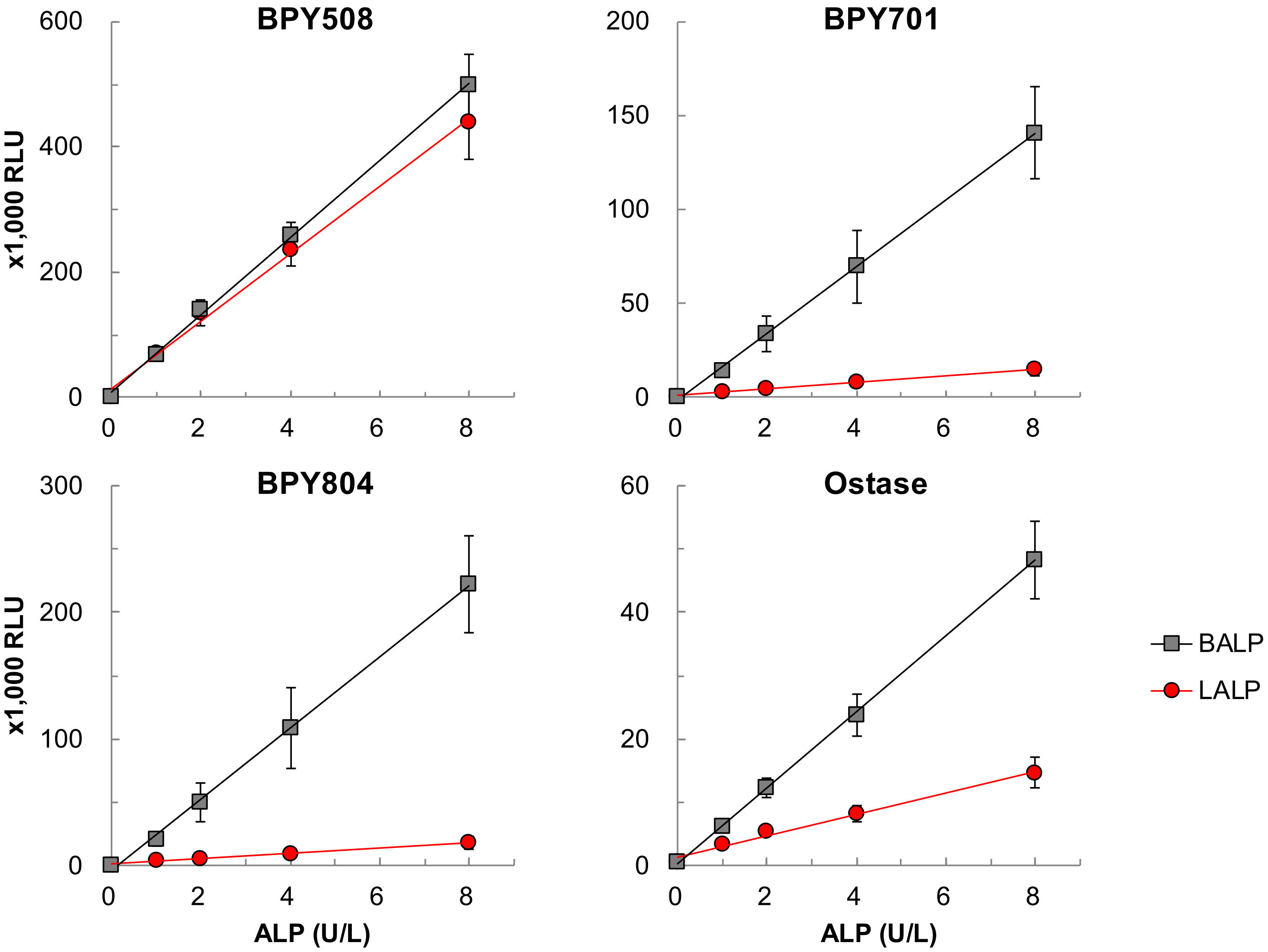

Fig. S1. Bone alkaline phosphatase (BALP) and liver alkaline phosphatase (LALP) reactivity in serially diluted serum specimens. Six BALP-dominant (B/BL ratio ≥ 90%) and six LALP-dominant (B/BL ratio < 10%) serum specimens were serially diluted to 8, 4, 2, and 1 U/L of alkaline phosphatase (ALP) activity, and their reactivity was evaluated using anti-mouse IgG-coated immunoassays with BPY508, BPY701, BPY804, or the Ostase antibody. Each immunoassay was performed in duplicate, and the mean value was used for analysis. The dots and error bars represent the mean ± SD of the six specimens.
