# Supplementary material for: Development of bone alkaline phosphatase-specific monoclonal antibodies and immunoassay exhibiting low cross-reactivity to liver isoform
Source: JBMR Plus. 2026 Apr 27;10(6):ziag080. doi: 10.1093/jbmrpl/ziag080 (PMC13184525; doi:10.1093/jbmrpl/ziag080)
Supplement: Fig_S2_ziag080 [file fig_s2_ziag080.pdf]

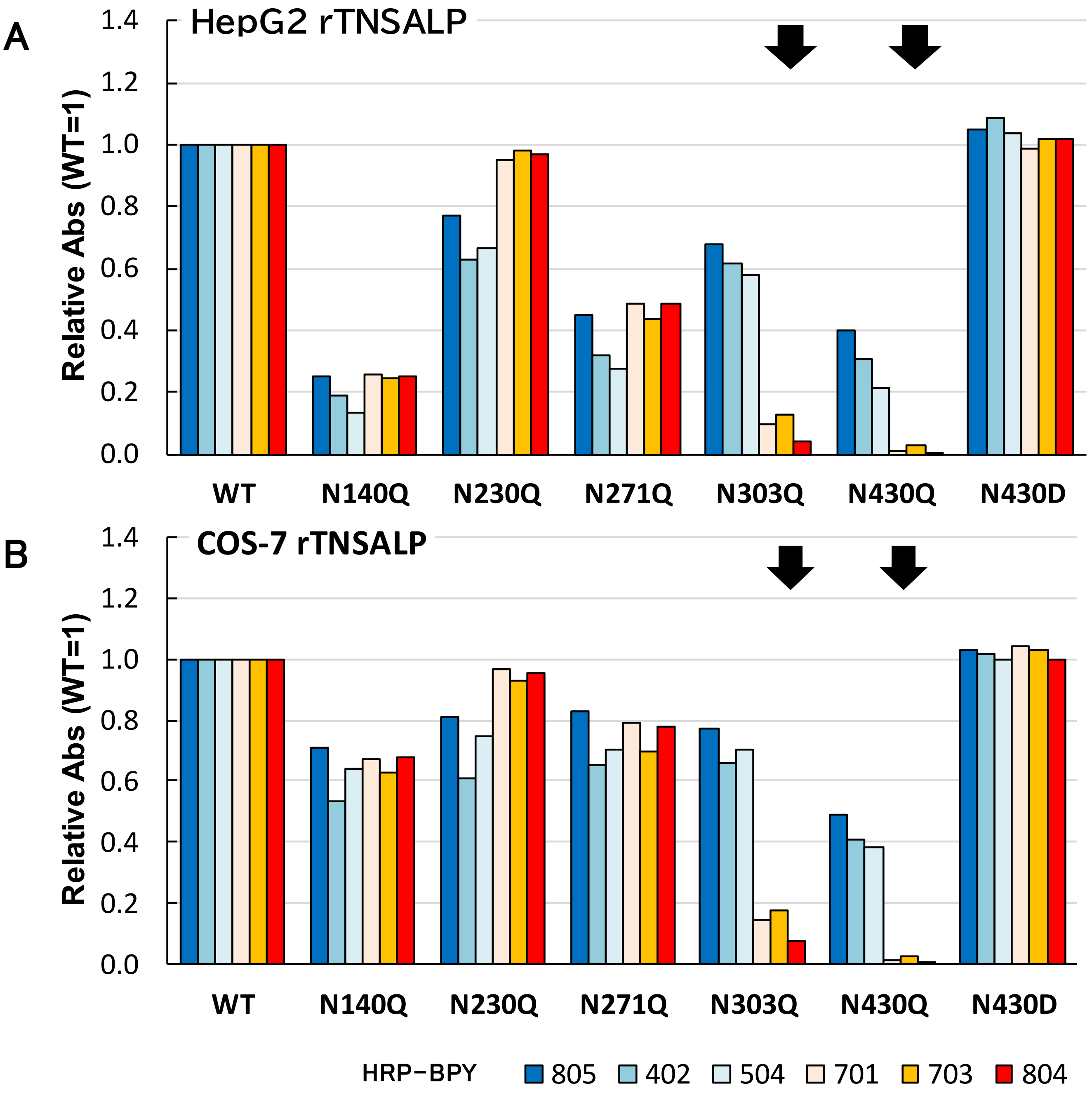

Fig. S2. Reactivity to N-glycosylation site mutants assessed by sandwich ELISA. Recombinant tissue-nonspecific alkaline phosphatase (rTNSALP) in culture supernatants was measured by sandwich ELISA with anti-FLAG capture antibody and horseradish peroxidase (HRP)-conjugated BPY detection antibodies. Reaction intensities are expressed as relative absorbance values normalized to wild-type (WT = 1), as indicated on the y-axis ["Relative Abs (WT = 1)"]. **A**: Culture supernatant from HepG2 cells (5× dilution). **B**: Culture supernatant from COS-7 cells (5× dilution). Arrows indicate reduced reactivity attributable to the mutations.
