# Supplementary material for: Development of bone alkaline phosphatase-specific monoclonal antibodies and immunoassay exhibiting low cross-reactivity to liver isoform
Source: JBMR Plus. 2026 Apr 27;10(6):ziag080. doi: 10.1093/jbmrpl/ziag080 (PMC13184525; doi:10.1093/jbmrpl/ziag080)
Supplement: Fig_S3_ziag080 [file fig_s3_ziag080.pdf]

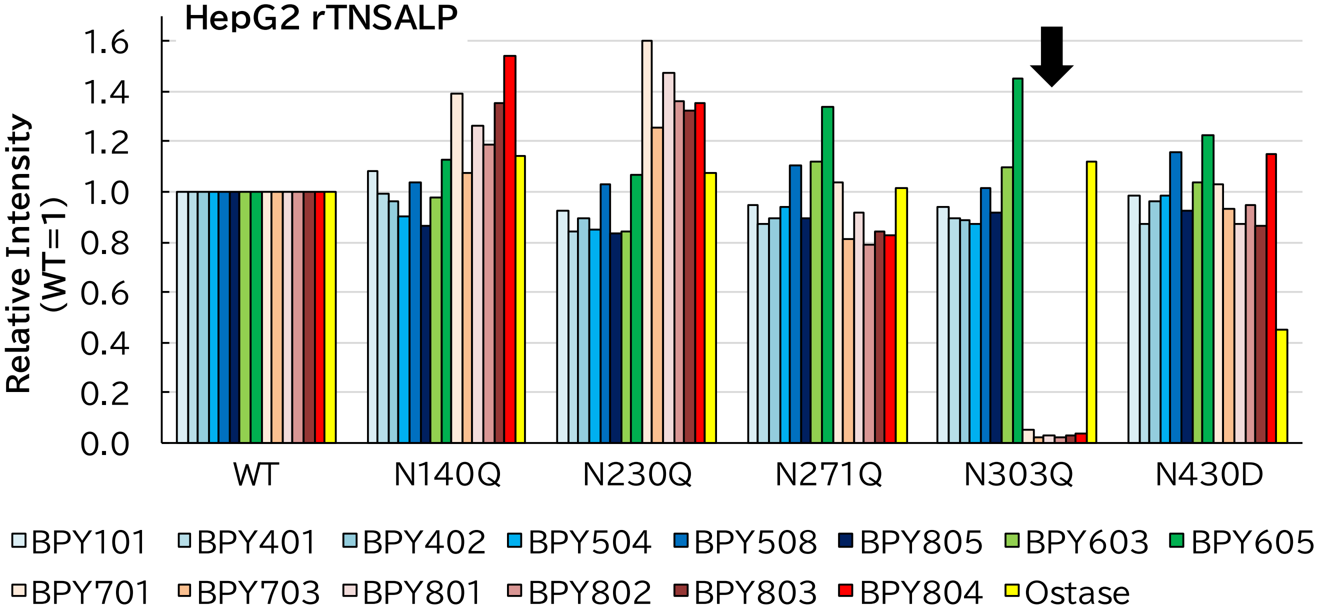

Fig. S3. Reactivity to N-glycosylation site mutants assessed by anti-mouse IgG-coated immunoassay. Recombinant tissue-nonspecific alkaline phosphatase (rTNSALP)-expressing culture supernatants of HepG2 cells (2 U/L of ALP activity) were analyzed using anti-mouse IgG-coated immunoassay. The mean reaction intensities (RLU) of duplicate assays are expressed as relative values normalized to the wild-type (WT = 1), as indicated on the y-axis [“Relative Intensity (WT = 1)”]. The arrow indicates reduced reactivity attributable to the mutation.
