# Supplementary material for: Development of bone alkaline phosphatase-specific monoclonal antibodies and immunoassay exhibiting low cross-reactivity to liver isoform
Source: JBMR Plus. 2026 Apr 27;10(6):ziag080. doi: 10.1093/jbmrpl/ziag080 (PMC13184525; doi:10.1093/jbmrpl/ziag080)
Supplement: Table_S1_ziag080 [file table_s1_ziag080.pdf]

Table S1. Cross-reactivity to liver alkaline phosphatase (LALP).

Antibody	Comparison	n	Mean diff	95% CI low	95% CI high	t	p (raw)	p (Holm)	Significant (0.05)
BPY504	BPY508	6	0.095	−0.142	0.333	1.032	0.349330	1.00000	FALSE
BPY401	BPY508	6	0.047	−0.094	0.188	0.851	0.433648	1.00000	FALSE
BPY402	BPY508	6	0.005	−0.114	0.124	0.108	0.917975	1.00000	FALSE
BPY508	baseline								
BPY805	BPY508	6	−0.013	−0.057	0.030	−0.790	0.465136	1.00000	FALSE
BPY101	BPY508	6	−0.106	−0.210	−0.003	−2.637	0.046142	0.23071	FALSE
BPY603	BPY508	6	−0.282	−0.349	−0.215	−10.819	0.000117	0.00070	TRUE
BPY605	BPY508	6	−0.387	−0.430	−0.345	−23.441	0.000003	0.00004	TRUE
BPY703	BPY508	6	−0.580	−0.698	−0.463	−12.679	0.000054	0.00049	TRUE
BPY802	BPY508	6	−0.625	−0.745	−0.504	−13.353	0.000042	0.00049	TRUE
BPY803	BPY508	6	−0.629	−0.754	−0.504	−12.893	0.000050	0.00049	TRUE
BPY801	BPY508	6	−0.648	−0.765	−0.532	−14.331	0.000030	0.00039	TRUE
BPY701	BPY508	6	−0.649	−0.776	−0.522	−13.129	0.000046	0.00049	TRUE
BPY804	BPY508	6	−0.659	−0.805	−0.513	−11.609	0.000083	0.00058	TRUE
Ostase	BPY508	6	−0.514	−0.612	−0.416	−13.433	0.000041	0.00049	TRUE

Cross-reactivity to LALP was calculated, based on the results in Table 1, as the reaction to each LALP-dominant specimen divided by the mean reaction of the bone alkaline phosphatase (BALP)-dominant specimens. Cross-reactivities in anti-mouse IgG-coated immunoassays with the 14 BPY antibodies and the Ostase antibody (Fig. 1) were compared using paired *t*-tests as planned contrasts with Holm adjustment against BPY508 (baseline, representing a typical nonselective antibody). CI, confidence interval.
