# Supplementary material for: Development of bone alkaline phosphatase-specific monoclonal antibodies and immunoassay exhibiting low cross-reactivity to liver isoform
Source: JBMR Plus. 2026 Apr 27;10(6):ziag080. doi: 10.1093/jbmrpl/ziag080 (PMC13184525; doi:10.1093/jbmrpl/ziag080)
Supplement: Table_S2_ziag080 [file table_s2_ziag080.pdf]

Table S2. Cross-reactivity to recombinant intestinal alkaline phosphatase (rIALP) and placental alkaline phosphatase (PALP).

	ALP isozyme (U/L)			ALP isozyme (U/L)	
	BALP	rIALP	PALP	rIALP	PALP
	10	1,000	1,000	1,000	1,000
mAb	RLU			Cross-Reactivity (%)	
BPY101	271,057	4,099	90,319	0.015	0.33
BPY401	762,352	888	70,085	0.001	0.09
BPY402	576,049	914	74,154	0.002	0.13
BPY504	704,792	904	44,505	0.001	0.06
BPY508	675,158	926	105,598	0.001	0.16
BPY603	693,957	1,280	84,016	0.002	0.12
BPY605	705,644	980	73,768	0.001	0.10
BPY701	71,039	2,054	4,869	0.029	0.07
BPY703	120,334	3,702	13,624	0.031	0.11
BPY801	85,560	2,411	6,322	0.028	0.07
BPY802	108,730	1,849	7,380	0.017	0.07
BPY803	113,360	1,304	7,765	0.012	0.07
BPY804	255,632	2,034	11,086	0.008	0.04
BPY805	1,043,144	1,369	276,558	0.001	0.27

Bone alkaline phosphatase (BALP), rIALP, and PALP were measured using anti-mouse IgG-coated immunoassays with the 14 BPY antibodies. Cross-reactivity was calculated as the reactivity (RLU) to rIALP or PALP (1,000 U/L) divided by the reactivity (RLU) to BALP (10 U/L).
